# DEEP OSCILLATION THERAPY ENHANCES EARLY REHABILITATION AFTER ACL RECONSTRUCTION: A RANDOMIZED CONTROLLED TRIAL

**DOI:** 10.2340/jrm.v57.44416

**Published:** 2025-10-07

**Authors:** Katarzyna OESTERVEMB, Robert TRYBULSKI, Elżbieta SZCZYGIEŁ, Anna SZCZYGIELSKA-BABIUCH, Bartłomiej KACPRZAK, Magdalena HAGNER-DRENGOWSKA

**Affiliations:** 1 Private Practice of Rehabilitation Fizjoactive, Kutno; 2 Medical Department of the Upper Silesian University in Katowice, Katowice; 3 Orthopedic Rehabilitation Institute, Faculty of Rehabilitation, University of Physical Education in Krakow, Krakow; 4 Specialist Hospital J. Dietl, Clinic of Rehabilitation, Krakow; 5 Cracow University of Health Promotion, Krakow; 6 Orto Med Sport, Łódźd; 7 Sport Research Center, Nicolaus Copernicus University, Torun, Poland

**Keywords:** physical therapy modalities, postoperative care, knee joint/physiopathology, pain threshold

## Abstract

**Objective:**

This study investigated the effectiveness of deep oscillation therapy on early rehabilitation outcomes and inflammatory markers in 60 patients (32.7 ± 6.5 years) following anterior cruciate ligament reconstruction.

**Design:**

A randomized controlled study was undertaken.

**Methods:**

Patients were randomly allocated into a deep oscillation therapy group (*n* = 30) receiving a physiotherapy protocol plus deep oscillation therapy, and a control group (*n* = 30) receiving a physiotherapy protocol alone. Outcomes assessed over 4 weeks included pain (algesimeter), knee circumference (swelling), active range of motion for flexion and extension (goniometer), and biomarkers: C-reactive protein (inflammation) and D-dimer (thromboembolic risk) blood tests.

**Results:**

Results showed significantly (*p* < 0.01) higher pressure pain threshold (i.e., increased tolerance of pressure-evoked pain), swelling reduction, and improved knee flexion and extension range of motion in the deep oscillation therapy compared with the control group after 2 and 4 weeks. The deep oscillation therapy achieved full extension by week four. Deep oscillation therapy also led to a more dynamic and pronounced decrease in C-reactive protein and D-dimer levels in the deep oscillation therapy compared with the control group (*p* < 0.01), with the deep oscillation therapy exhibiting significantly lower levels after 2 and 4 weeks. Correlations were observed in the deep oscillation therapy between reduced inflammatory markers and improved mobility and swelling.

**Conclusions:**

These findings suggest that deep oscillation therapy can significantly enhance early rehabilitation outcomes and reduce inflammation in patients after anterior cruciate ligament reconstruction.

## LAY ABSTRACT

After surgery to repair the anterior cruciate ligament, patients often deal with pain, swelling, and limited movement. This study looked at whether a gentle treatment called deep oscillation therapy could help people recover faster. Sixty patients were divided into 2 groups: 1 received regular physiotherapy, and the other was given the same therapy plus deep oscillation therapy. Over 4 weeks, those who received deep oscillation therapy had less pain and swelling, could move their knee better, and showed signs of less inflammation in their bodies. These improvements happened more quickly and were more noticeable than in those who received regular therapy alone. By the end of the study, patients using deep oscillation therapy had better flexibility and could straighten their knees fully. These results suggest that adding deep oscillation therapy to regular rehabilitation could speed up recovery and improve outcomes after anterior cruciate ligament surgery, helping people return to their daily activities sooner and more comfortably.

Anterior cruciate ligament (ACL) rupture is a common and debilitating knee injury, common to both athletes and physically active individuals, as well as the general population, often necessitating surgical reconstruction to restore knee stability and function (1). While advancements in surgical techniques have improved outcomes, the early postoperative period is frequently characterized by significant pain, swelling, limited range of motion (ROM), and a pronounced inflammatory response (2, 3). These factors can impede the initiation and progression of early rehabilitation, potentially delaying functional recovery and return to activity (4, 5). Effective management of postoperative inflammation, pain, and oedema is crucial for optimizing early rehabilitation outcomes and preventing complications such as arthrofibrosis and delayed muscle activation (6, 7).

Deep oscillation therapy (DOT) is a non-invasive and non-traumatic therapeutic modality that utilizes intermittent electrostatic attraction and repulsion to generate biologically effective oscillations within the treated tissue (8, 9). Unlike other forms of therapy that rely on external pressure (e.g., manual therapy, compression, percussive massage), DOT’s effect takes place directly within the tissue, penetrating up to 8 cm deep into various layers, including skin, connective tissue, subcutaneous fat, muscles, blood, and lymph vessels by promoting the transportation of interstitial fluids (9, 10), DOT may support the clearance of inflammatory mediators and accelerate recovery (9, 11).

DOT has been shown to promote muscle relaxation and improve tissue elasticity (9, 11). Recent clinical evidence and reviews highlight the increasing use of DOT in various medical fields (14), including postoperative pain management (15), sports injuries (12), and chronic pain management (16); however, with some contradictory results. For instance, in 1 study (16) focusing on the effects of DOT in active individuals with and without lower-leg pain, it was observed that while DOT significantly reduced pain levels, it did not lead to clinically meaningful improvements in ankle dorsiflexion range of motion. On the other hand, in 1 study (17) investigating early rehabilitation after knee joint arthroplasty, it was observed that patients who received DOT alongside kinesiotherapy experienced significantly greater reductions in pain and swelling, as well as improved range of motion, compared with those receiving a physiotherapy protocol. These mixed findings suggest that while DOT may have benefits in certain contexts, its effects on specific clinical and biochemical outcomes during post-surgical rehabilitation remain insufficiently understood. This indicates a research gap in understanding the specific clinical and biochemical effects of DOT during post-surgical rehabilitation.

Importantly, pain levels and swelling are key clinical indicators of recovery, but they provide only a partial view of the underlying biological processes (18). Inflammatory markers, such as C-reactive protein and D-dimer, reflect systemic and localized inflammatory responses, which play a critical role in tissue healing and the risk of complications such as vascular embolism. By incorporating the analysis of these biochemical markers alongside traditional clinical outcomes, we can capture a more comprehensive picture of patient recovery. This holistic approach is an advance in DOT research, moving beyond symptom management to understand how DOT may influence the physiological processes that underpin healing and rehabilitation. We hypothesize that the application of DOT will lead to superior clinical and biochemical outcomes, while acknowledging that the modest sample size provides preliminary but valuable insights.

## METHODS

### Patients and setting

Patients were recruited from the Orthopedic Surgery Department in Krakow (Poland) between 2024 and 2025. Eligible participants were identified by clinical staff based on predefined inclusion and exclusion criteria. Patients scheduled for arthroscopic ACL reconstruction were informed about the study during their preoperative consultations. A member of the research team explained the purpose, procedures, and voluntary nature of the study. All participants were fully informed about the nature and scope of the interventions and provided written informed consent prior to participation.

### Trial design and randomization

This study was designed as a prospective, parallel-group randomized controlled trial with an allocation ratio of 1:1. The trial was reported in accordance with the TIDieR checklist to ensure a transparent description of the intervention. Ethical approval was obtained from the Bioethics Committee of the Medical Chamber in Krakow (No. 128/KBL/OIL/2018). Patients were recruited and initially treated in the orthopaedic surgery inpatient ward and subsequently continued their rehabilitation in an outpatient physiotherapy setting. All therapeutic procedures were delivered by licensed physiotherapists trained in accordance with the manufacturer’s guidelines for DOT. In the event of intolerance of DOT (e.g., excessive discomfort, local skin irritation, or inability to tolerate the procedure), participants would have been withdrawn from the intervention while continuing the standard physiotherapy protocol; however, no such cases occurred during the study.

### Eligibility criteria

Participants were eligible for inclusion if they were between 25 and 40 years of age and had undergone arthroscopic ACL reconstruction using the double-bundle technique with semitendinosus and gracilis (STGR) muscle grafts, stabilized with the Rigidfix system (DePuy Synthes, Raynham, MA, USA). Additional inclusion criteria required the ability to begin the proposed rehabilitation protocol from baseline (postoperative day 3) and the absence of vascular diseases, including deep vein thrombosis, postphlebitic syndrome, or skin atrophy. Participants were also required to have no systemic contraindications to early postoperative rehabilitation. Exclusion criteria included the inability to tolerate deep oscillation therapy (for the DOT group) or the early rehabilitation exercises, failure to meet any of the inclusion criteria, or unwillingness to participate in or continue with the study.

Patients were recruited among individuals scheduled for ACL reconstruction surgery. Recruitment was conducted through direct clinical referral by orthopaedic surgeons and healthcare staff during routine preoperative evaluations. Eligible patients were approached postoperatively by a member of the research team, who explained the study protocol and obtained consent. No advertisements or self-referral methods were used for recruitment.

### Intervention and comparator

The trial compared 2 rehabilitation approaches following arthroscopic ACL reconstruction: a physiotherapy protocol rehabilitation programme (comparator) and the same physiotherapy protocol extended by the additional application of DOT (intervention).

*A physiotherapy protocol for rehabilitation (comparator).* A physiotherapy protocol programme aimed to restore mobility, reduce pain and swelling, and promote functional recovery of the operated knee. Rehabilitation began at baseline (postoperative day 3) and lasted for 4 weeks. The programme included the following components: Week 1: Elevation of the operated limb; passive extension exercises; gradual, pain-free increase in knee flexion (up to 60°); self-assisted isometric exercises for thigh and lower leg muscles; walking with two crutches. At 2 weeks: Passive exercises focusing on extension and increased flexion (up to 90–100°); continued isometric thigh and lower leg exercises; multidirectional patella and scar mobilization; active exercises; walking with one crutch; post-isometric muscle relaxation; proprioception exercises on stable surfaces; partial squats with knee flexion up to 60°. Weeks 3 and 4: Gradual increase in knee flexion (up to 120°); proprioception exercises on both stable and unstable surfaces; continuation of all previous exercises.

All exercises were designed to the participant’s pain tolerance and progression, with close monitoring by a physical therapist. No additional physical or informational materials beyond standard clinical guidance were used.

*Deep oscillation therapy (intervention).* Participants in the intervention group received the same physiotherapy protocol rehabilitation described above, with the addition of deep oscillation therapy administered to the knee joint. Deep oscillation treatment was performed using a CE-certified device (PHYSIOMED ELEKTROMEDIZIN AG, Germany) applied 3 times per week for 4 weeks, totalling 12 sessions. Each session lasted 40 min: 20 min on the anterior (front) side and 20 min on the posterior (back) side of the operated limb. During treatment, patients wore sportswear enabling direct access to the knee. The patient was first positioned supine for anterior treatment and then prone for posterior treatment. The applicator, an active electrode with a 95 mm circumference, was moved over the skin surface while a passive electrode was held in the patient’s hand to complete the electrical circuit. DOT was applied with a CE-certified device (PHYSIOMED ELEKTROMEDIZIN AG, Germany), and noted that DOT is not yet part of formal guidelines but is supported by clinical evidence

The treatment protocol consisted of 3 parts: Part I: 10 min at 120–180 Hz, Part II: 5 min at 14–30 Hz, Part III: 5 min at 85 Hz. The deep oscillation therapy was individualized to each participant by adjusting the applicator movement and frequency according to tolerance and treatment guidelines. No formal instruction manuals were distributed, but therapists administering the intervention were trained according to manufacturer protocols.

*Concomitant care and modifications.* Both groups received the same standard postoperative care, including pain management and surgical follow-up. No rescue interventions or additional therapies (e.g., pharmacological treatments targeting inflammation) were permitted during the trial that could interfere with the rehabilitation protocols. Participants unable to tolerate DOT were to be withdrawn from the intervention; however, no such discontinuations occurred during the study.

*Adherence and fidelity.* All patients completed the full 4-week rehabilitation programme, including all deep oscillation sessions for the intervention group (DOT *n* = 30, CG *n* = 30). Treatment fidelity was ensured by trained physical therapists following standardized protocols. Session attendance was recorded, and therapists monitored participant adherence and response throughout. Participants were classified as treated as planned if they completed at least 90% of prescribed sessions. All participants met this threshold.

### Outcomes

**Fig. 1:**
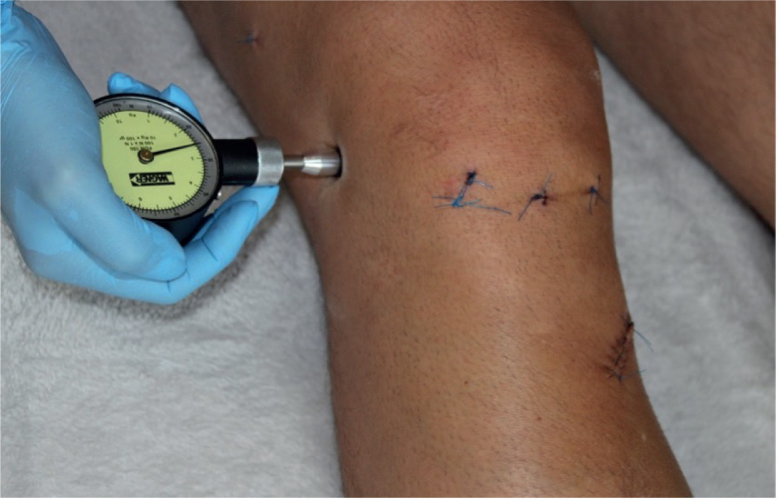
Pressure measurement with an algesimeter around the lateral compartment of the knee joint at the level of the joint space (own source).

*Primary outcome*. The primary outcome of the trial was pressure pain threshold around the operated knee joint (Fig. 1). Pressure pain threshold was measured using a pressure algesimeter (WAGNER INSTRUMENTS, Greenwich, CT, USA, model FORCE DIAL FDN 100) applied to the lateral compartment of the knee at the joint space level. This method quantifies the mechanical pressure (in N/cm²) required to elicit pain. The analysis measure was the change in pain threshold from baseline (postoperative day 3), and results were aggregated as mean values ± standard deviation within each group.

Pain assessments were performed by trained physical therapists at 3 predefined timepoints: baseline (postoperative day 3) post-surgery, 2 weeks after baseline (postoperative day 3), and 4 weeks after baseline (postoperative day 3). This outcome was selected due to its clinical relevance in early postoperative ACL rehabilitation and its clinical interpretability in evaluating intervention efficacy. Although not drawn from a formal core outcome set, pain reduction is a widely accepted endpoint in post-ACL surgery rehabilitation trials[22].

### Secondary outcomes

1. *Knee swelling*. Knee swelling was assessed as a secondary outcome through the measurement of limb circumference at the level of the knee joint gap. This was done using a non-elastic, flexible measuring tape marked in millimetres to ensure precise readings. All measurements were performed by a trained physiotherapist, who followed a standardized protocol for anatomical landmark identification and patient positioning. Patients were placed in a relaxed, supine position with the lower limbs extended and muscles relaxed. The measurement site was located by palpating the joint line of the knee – specifically, the space between the femoral condyles and the tibial plateau on the lateral aspect of the joint. Circumference was measured horizontally around the leg at this level, ensuring that the tape lay flat against the skin without compressing the soft tissues. Each measurement was taken 3 times, and the mean value was recorded. Assessments were conducted at 3 timepoints: on the third day after surgery (baseline: postoperative day 3), at 2 weeks, and at 4 weeks post-baseline (postoperative day 3). The analysis metric for knee swelling was the change in circumference (in centimetres) from baseline (postoperative day 3) values. Group data were aggregated and expressed as mean ± standard deviation.

2. *Active range of motion (ROM)*. Active range of motion (ROM) of the operated knee was assessed as a 2-weekly outcome, specifically measuring both flexion and extension. Measurements were conducted using a standard universal plastic goniometer with a 360° range and 1° increments. All assessments were performed by a licensed physiotherapist trained in orthopaedic evaluation techniques. To ensure consistency and reduce variability, the same therapist measured ROM for each participant throughout the study. For each measurement, the patient was positioned supine on a flat rehabilitation table with the hip in a neutral position. Flexion was measured by instructing the patient to actively bend the knee as far as possible within a pain-free range. The goniometer was aligned with the lateral epicondyle of the femur as the axis, the stationary arm along the lateral midline of the femur towards the greater trochanter, and the moving arm along the lateral midline of the fibula towards the lateral malleolus. Extension was assessed with the patient remaining supine, with the heel supported to allow full passive extension. The patient was instructed to actively extend the knee, and the same goniometric landmarks were used. Each measurement was taken 3 times, and the highest value achieved without pain was recorded as the final result. Assessments were performed at 3 timepoints: at baseline (postoperative day 3), at 2 weeks, and at 4 weeks following baseline (postoperative day 3). To standardize conditions, ROM was always assessed before the daily rehabilitation session to avoid temporary mobility increases caused by exercise. The primary analysis measure was the change in degrees of active flexion and extension from baseline (postoperative day 3), with group-level results reported as mean ± standard deviation.

3. *Active inflammatory markers*. Blood samples were collected at 3 standardized timepoints: baseline (postoperative day 3), 2 weeks, and 4 weeks post-baseline (postoperative day 3). Sampling was conducted by a trained nurse in a controlled clinical setting. Capillary blood was obtained via a finger prick using a sterile lancet, following standard aseptic technique. A small volume of blood (approximately 50 µL) was collected into test-specific cartridges compatible with the Nano-Checker 710V diagnostic device (version 1) (Nano-Ditech Corporation, Cranbury, NJ, USA). This portable, point-of-care immunoassay system is designed for rapid, semi-quantitative detection of inflammatory and coagulation markers.

### Blood

CRP values (mg/L) were expressed in milligrams, and D-dimer (μg/mL FEU) levels were reported in micrograms per millilitre. The Nano-Checker analyser provided digital readouts within minutes, ensuring prompt and reliable data capture. Each test was performed according to the manufacturer’s instructions, and all equipment was calibrated prior to use. The same device and test method were used for all participants throughout the study to ensure consistency. The analysis measure for inflammatory markers was the absolute value at each time point, as well as the change from baseline (postoperative day 3). Data were aggregated as mean ± standard deviation for each study group.

### Harms

Adverse events were monitored throughout the study period using both systematic (active) and non-systematic (passive) methods. Given the non-invasive nature of the interventions – a physiotherapy protocol and deep oscillation therapy – serious adverse events were not anticipated; however, structured procedures were in place to ensure the detection and documentation of any harms.

Systematic monitoring was carried out by the treating physiotherapist during each rehabilitation session, which occurred 3 times per week over the 4-week intervention period. At each session, participants were asked about new or worsening symptoms, such as excessive post-exercise pain, swelling beyond expected levels, local skin irritation, or unusual discomfort during or after treatment. These symptoms were documented using a standardized symptom checklist developed for the study. The physiotherapist also visually inspected the treated area for signs of irritation or inflammation. The presence, frequency, and duration of any harms were recorded, and data were later aggregated as the number and percentage of participants affected in each group. As the physiotherapists were delivering the interventions, they were not blinded to group allocation.

In addition to active surveillance, non-systematic (passive) harm reporting was encouraged. Participants were instructed to report any unexpected symptoms or concerns occurring between sessions, either in person during the next visit or remotely via phone or email. All reported harms were reviewed and coded by a clinical member of the research team, who was also unblinded. Each harm was categorized by severity (mild, moderate, or severe), seriousness (non-serious or serious), body system affected, and its likely relationship to the intervention (unrelated, possibly related, or probably related). Although no formal coding system was used, grading was informed by principles from the Common Terminology Criteria for Adverse Events (CTCAE v5.0). No serious adverse events occurred during the study.

### Sample size

The sample size calculation was based on the primary outcome, which was the change in pain sensitivity around the operated knee joint, assessed via pressure algesimeter. Because the data were analysed using non-parametric methods – specifically the Mann–Whitney *U* test for between-group comparisons and the Friedman test for repeated measures – a sample size estimation appropriate for non-parametric testing was conducted. Given the lack of precise prior data on pain threshold distributions in this population, a moderate effect size (rank-based) corresponding approximately to a probability of superiority (AUC) of 0.64 between groups was assumed, which is analogous to a Cohen’s *d* of around 0.5–0.7. To detect this effect with a 2-sided significance level (α) of 0.05 and power (1–β) of 0.80, a minimum of 26 participants per group was estimated to be sufficient based on sample size tables and simulations for the Mann–Whitney *U* test. To account for possible dropouts, missing data, or protocol deviations, the planned sample size was increased by 15%, resulting in 30 participants per group and a total sample size of 60 participants. Sample size calculations were performed using G*Power software (version 3.1.9.2; https://www.psychologie.hhu.de/arbeitsgruppen/allgemeine-psychologie-und-arbeitspsychologie/gpower), applying parameters appropriate for non-parametric two-group comparisons.

### Statistical analysis method

Statistical analyses were conducted using the PQStat statistical software (version 1.8.2.230; https://pqstat.pl/). Between-group comparisons of outcome measures at each time point were performed using the Mann–Whitney *U* test due to the non-normal distribution of data. To evaluate changes over the 3 measurement periods within each group, the Friedman test for repeated measures was applied, followed by post-hoc Dunn’s test with Bonferroni correction for multiple comparisons. Statistical significance was defined as a *p*-value less than 0.05, while *p*-values below 0.01 were considered highly significant. Descriptive statistics for continuous variables included the arithmetic mean (x̄), standard deviation (SD), range (minimum to maximum), median (Me), as well as the first (Q1) and third quartiles (Q3). All analyses were 2-tailed, and assumptions for non-parametric tests were verified prior to analysis. Correlations between variables were assessed using Spearman’s rank correlation coefficient (ρ) with 2-tailed significance. All data were collected on standardized case report forms, entered into a secure electronic database, and verified by double data entry. Access was restricted to study investigators. Data were anonymized prior to analysis.

## RESULTS

### Participant flow

**Fig. 2:**
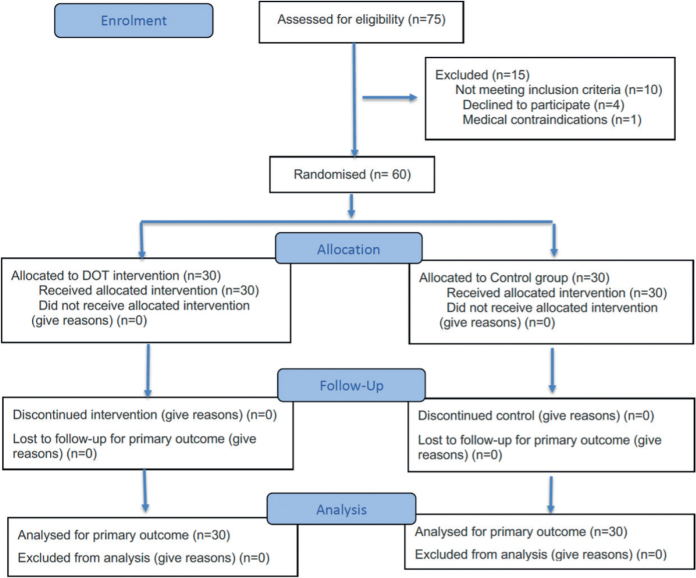
CONSORT 2025 flow diagram (19).

A total of 75 patients were initially evaluated for potential enrolment in the study (Fig. 2). Of these, 15 patients were excluded prior to randomization due to not meeting the inclusion criteria (*n* = 10), declining to participate (*n* = 4), and medical contraindications (*n* = 1). The remaining 60 eligible participants were randomly assigned in a 1:1 ratio to either the study group (DOT, *n* = 30) receiving a physiotherapy protocol plus deep oscillation therapy, or the control group (CG, *n* = 30) receiving a physiotherapy protocol alone. The average age of the participants was 32.7 ± 6.5 years (DOT: 29.45 ± 3.25 years; CG: 33.75 ± 2.5 years). Women accounted for 26.6% of the total sample, with men comprising 73.3%. All participants in both groups received the intervention as allocated. Furthermore, all patients completed the intervention protocol and the planned follow-up assessments. Therefore, the primary outcome analysis included all 60 participants (DOT = 30; CG = 30), indicating no loss to follow-up or attrition. Sex distribution by group was comparable due to stratified randomization.

### Primary outcome

The average pain sensation was measured using an algesimeter around the lateral compartment of the knee joint. In the DOT, mean pain threshold values were 14.07 ± 1.76 N at the baseline (postoperative day 3), increasing to 33.5 ± 6.18 N at 2 weeks, and 44.17 ± 5.53 N at 4 weeks. In the CG, mean values were 14.03 ± 1.96 N after the first measurement, 17.23 ± 3.27 N after 2 weeks, and 21.37 ± 3.76 N after the third (Table I). Significant correlations between the2 groups were observed at the 2- and 4-week time points (*p* < 0.01), with the DOT demonstrating notably pain threshold values, indicating pressure pain threshold (i.e., increased tolerance to pressure-evoked pain) following deep oscillation therapy. Both groups showed significant increases in pain threshold over time (*p* < 0.01), but the rate and magnitude of improvement were substantially greater in the DOT (Table I).

**Table I:** Results of pain assessments with the use of algesimeter [N]

Descriptive statistics		3rd day	2 weeks	4 weeks	Friedman test
Deep oscillation therapy group					
x±sd		14.07±1.76	33.50±6.18	44.17±4.56	T = 59.5126
R_min–max_		10–20	20–40	35–50	*p* < 0.0001
Q1		13.00	30.00	40.00	
Median		15.00	35.00	45.00	
Q3		15.00	40.00	45.00	
Homogeneous groups^1^		a	b	c	–
Control group					
x±sd		14.03±1.96	17.23±3.27	21.37±3.76	T = 48.5347
R_min–max_		10–18	13–25	15–30	*p* < 0.0001
Q1		13.00	15.00	20.00	
Median		15.00	15.00	20.00	
Q3		15.00	20.00	25.00	
Homogeneous groups^1^		a	b	c	–
Mann–Whitney *U* test	Z	0.297	6.4916	6.7565	–
	p	0.7665	< 0.0001	< 0.0001	–

1 Homogeneous groups of Friedman one-way repeated measure analysis from Dunn’s post-hoc test with Bonferroni correction.

### Secondary outcomes

Postoperative swelling was measured around the knee joint gap. At the first measurement, the mean circumference was 42.6 ± 2.36 cm in the DOT and 43.47 ± 2.41 cm in the CG. After 2 weeks of additional deep oscillation therapy, the circumference in the DOT decreased to 39.15 ± 2.03 cm, while in the CG it reduced to 42.47 ± 2.47 cm. The final measurement showed a further decrease, with the DOT averaging 37.62 ± 1.79 cm and the CG 41.38 ± 2.47 cm (Table II).

**Table II:** Metric oedema measurement in the operated lower limb. taken through the knee joint (cm)

Descriptive statistics		3rd day	2 weeks	4 weeks	Friedman test
Deep oscillation therapy group					
x±sd		42.6±2.36	39.15±2.03	37.62±1.79	T = 53.0667
R_min–max_		36.5–48	35–43.5	33–44	*p* < 0.0001
Q1		41.25	38.00	37.00	
Median		43.00	39.00	38.00	
Q3		44.00	41.00	38.00	
Homogeneous groups^1^		c	b	a	–
Control group					
x±sd		43.47±2.41	42.47±2.43	41.38±2.47	T = 58.5128
R_min–max_		39–47	37–45	35–44	*p* < 0.0001
Q1		42.63	41.63	40.25	
Median		44.25	43.50	42.00	
Q3		44.88	44.00	43.00	
Homogeneous groups^1^		c	b	a	–
Mann–Whitney *U* test	Z	1.955	4.6535	4.9567	–
	p	0.0506	< 0.0001	< 0.0001	–

1 Homogeneous groups of Friedman One-Way Repeated Measure analysis from Dunn’s post-hoc test with Bonferroni correction.

At baseline (postoperative day 3), the average knee flexion range in the operated joint was 17.3 ± 10.59° for the DOT and 7.03 ± 7.11° for the CG. After 2 weeks of additional deep oscillation treatment, the range of motion increased to 64.5 ± 7.81° in the DOT, compared with 38.97 ± 7.81° in the CG. At 4 weeks post-surgery, flexion measurements averaged 92.83 ± 9.89° in the DOT and 53.83 ± 6.54° in the CG.

A significant correlation (*p* < 0.05) between the 2 groups was observed at baseline (postoperative day 3). This significance increased at both the 2- and 4-week time points (*p* < 0.01), consistently favouring the DOT. Both groups showed significant improvements in flexion range over time (*p* < 0.01); however, the increase was markedly greater in the experimental group.

Regarding knee extension, at baseline (postoperative day 3), the mean extension deficit was 6.9 ± 2.34° for the DOT and 6.37 ± 4.24° for the CG. After 2 weeks of treatment, the extension deficit decreased to 2.03 ± 2.31° in the DOT, while it remained around 6.4 ± 1.65° in the CG. At 4 weeks, the DOT achieved full extension (0 ± 0°), whereas the CG averaged an extension deficit of 5.67 ± 1.81° (Table III).

**Table III:** Measurement results of extension and flexion in the operated knee joint [o]

Descriptive statistics		Knee joint extension				Knee joint flexion			
		3rd day	2 weeks	4 weeks	Friedman test	3rd day	2 weeks	4 weeks	Friedman test
Deep oscillation therapy group									
x±sd		6.90±2.34	2.03±2.31	0±0	T = 52.16	17.30±10.59	64.50±7.81	92.83±9.89	T = 60.0000
R_min–max_		4–10	0–5	0–0	*p* < 0.0001	0–40	50–80	65–110	*p* < 0.0001
Q1		5.00	0.00	0.00		10.00	60.00	90.00	
Median		5.50	0.00	0.00		20.00	65.00	95.00	
Q3		10.00	5.00	0.00		25.00	70.00	98.75	
Homogeneous groups^1^		b	a	a		a	b	c	
Control group									
x±sd		6.37±4.24	6.40±1.65	5.67±1.81	T = 4.4719	7.03±7.11	38.97±6.65	53.83±6.54	T = 60.0000
R_min–max_		0–15	5–10	3–10	*p* = 0.1069	0–25	30–55	40–65	*p* < 0.0001
Q1		3.25	5.00	5.00		0.00	35.00	50.00	
Median		6.00	6.00	5.00		6.00	40.00	55.00	
Q3		8.75	7.00	5.00		10.00	45.00	60.00	
Homogeneous groups^1^		a	a	a		a	b	c	
Mann–Whitney *U* test	Z	0.88	5.9938	7.286	3.7082	6.6231	6.689
	p	0.3789	< 0.0001	< 0.0001	0.0002	< 0.0001	< 0.0001

1 Homogeneous groups of Friedman one-way repeated measure analysis from Dunn’s post-hoc test with Bonferroni correction.

No significant correlation (*p* > 0.05) between groups was found on the third day post-surgery for extension. However, significant correlations emerged at the 2- and 4-week measurements (*p* < 0.01), with the DOT showing better outcomes. The DOT demonstrated highly significant improvements in extension range over time (*p* < 0.01), while changes in the CG were not statistically significant (*p* > 0.05).

The CRP index was the first inflammatory parameter evaluated. After baseline (postoperative day 3), the CRP index totalled 34.39 ± 51.02 μg/ml in DOT and 26.78 ± 15.81 μg/ml in CG. After 2 weeks, the CRP index decreased fivefold to the level of 6.26 ± 10.91 μg/ml in DOT. In comparison, for the CG the level was 15.41 ± 9.26 μg/ml. After 4 weeks of the programme, the average CRP test results in DOT decreased to 1.84 ± 1.09 μg/ml, while in CG they amounted to 7.26 ± 5.07 μg/ml. At baseline (postoperative day 3), no significant (*p* > 0.05) correlation was found between DOT and CG. Nevertheless, after 2 and 4 weeks the correlations between the 2 groups proved to be highly significant (*p* < 0.01), with the lower results applying to DOT. In both groups, significant correlations (*p* < 0.01) were observed in the CRP test results depending on the measurement date, i.e., a decrease in the results was observed in both groups; nonetheless, in DOT it turned out to be much more dynamic (Table IV).

**Table IV:** Results of CRP (mg/L) and D-dimer (μg/mL FEU) in blood tests

Descriptive statistics		CRP				D-dimer			
		3rd day	2 weeks	4 weeks	Friedman test	3rd day	2 weeks	4 weeks	Friedman test
Deep oscillation group									
Arithmetic mean		34.39±51.02	6.26±10.91	1.84±1.09	T = 40.2000	323.76±331.16	255.74±226.24	138.90±123.89	T = 9.6571
Minimum		1.47–295	0–48.85	0–3.89	*p* < 0.0001	0–1408.34	0–942.6	0–352.78	*p* = 0.0080
Lower quartile		17.30	1.56	1.34		200.00	50.00	0.00	
Median		25.59	2.47	1.98		241.13	228.33	200.00	
Upper quartile		35.73	6.12	2.45		458.25	302.06	200.00	
Homogeneous groups^1^		b	a	a		b	b	a	
Control group									
Arithmetic mean		26.78±15.81	15.41±9.26	7.26±5.07	T = 31.2667	323.82±319.82	433.44±224.91	290.85±165.18	T = 14.5565
Minimum		1.00–53.2	1.90–31.2	1.34–17.53	*p* < 0.0001	0–1200.38	200.00–990.09	0–637.65	*p* = 0.0007
Lower quartile		11.69	5.81	3.37		200.00	265.75	200.00	
Median		28.42	14.87	5.46		203.28	360.23	277.79	
Upper quartile		39.66	23.45	10.44		452.66	487.55	438.36	
Homogeneous groups^1^		b	b	a		a	b	a	
Mann–Whitney *U* test	Z	0.1405	4.4292	4.9912		0.1348	3.2051	3.8141	
	p	0.8883	< 0.0001	< 0.0001	0.8928	0.0014	0.0001

1 Homogeneous groups of Friedman one-way repeated measure analysis from Dunn’s post-hoc test with Bonferroni correction.

Second, the D-dimer level was measured. The blood test performed on the 3rd day after surgery gave an average result of 323.76 ± 331.16 μg/ml for DOT and 323.82 ± 319.82 μg/ml for CG. Just after 2 weeks of implementing deep oscillation treatment additionally, the tested inflammatory index dropped to mean values of 255.74 ± 226.24 μg/ml while, at the same time, it increased to 433.44 ± 224.91 μg/ml in CG. After 4 weeks of the physiotherapy programme, the index began to decrease in both groups: in DOT to the level of 138.9 ± 123.89 μg/ml and in CG to the level of 290.85 ± 165.18 μg/ml.

At baseline (postoperative day 3), no significant (*p* > 0.05) correlation was found between the study and control groups. Nevertheless, after 2 and 4 weeks, the correlations between the groups were highly significant (*p* < 0.01), with the lower results concerning DOT. At first, a significant increase occurred in the CG, which was then followed by a significant decrease, but after 4 weeks the results turned out to be still relatively high. In contrast, no increase in DOT was found but only a slow decrease, and after 4 weeks the results were significantly lower than the initial ones (see Table IV).

In terms of increasing the mobility of the knee joint, a significant correlation (*p* < 0.001) was noted between the range of flexion and extension and the CRP protein level. The lower the inflammatory index, the greater the range of motion in the patients in DOT. In turn, in CG, this correlation was not so strong and turned out to be statistically insignificant. The situation was similar after 4 weeks of physiotherapy; however, during this time equally significant correlations (at the level of *p* < 0.05) were observed between the level of the CRP inflammatory index and the range of motion in the operated knee joint. Nevertheless, a stronger correlation related to DOT.

Additionally, a statistically significant correlation was noted between the D-dimer level and the range of motion in the operated knee joint in DOT after 2 weeks of the programme, while in CG it turned out to be weak and insignificant. However, after 4 weeks, in both groups statistically significant correlations were observed between the level of D-dimer and the range of motion in the operated joint. Stronger correlations appeared in the group with deep oscillation therapy applied. Moreover, it was detected that between the oedema measurement results after 2 weeks, there was a significant mean strength relationship between the oedema circumference and the CRP level in DOT ρ = –0.557, *p* < 0.05. This was not observed in CG, where the correlation was weak and non-significant ρ = –0.227, *p* > 0.05. Nonetheless, after 4 weeks of physiotherapy, there were significant correlations of moderate strength in both groups, with the more significant ones referring to DOT ρ = –0.598, *p* < 0.05. In CG ρ = –0.417, *p* < 0.05.

An analogous situation concerned the correlation between oedema measurements and D-dimer levels. In DOT, after 2 weeks ρ = –0.622 *p* < 0.001, after 4 weeks ρ = –0.557, *p* < 0.001. For the CG, the indicators totalled as follows: after 2 weeks ρ = –0.339, *p* > 0.05, after 4 weeks ρ = –0.489, *p* < 0.05.

## DISCUSSION

This study aimed to evaluate the therapeutic potential of DOT as an adjunct to a physiotherapy protocol in the early postoperative rehabilitation following anterior ACL reconstruction. The results showed that the additional application of DOT significantly enhances clinical recovery including pain reduction, oedema control, joint mobility, and biochemical markers of inflammation and thrombosis risk. Compared with a physiotherapy protocol alone, patients receiving DOT exhibited more rapid and pronounced improvements in pain thresholds, greater reductions in knee joint swelling, and superior gains in both flexion and extension range of motion. Furthermore, DOT was associated with a steeper decline in C-reactive protein (CRP) and D-dimer levels, indicating a stronger anti-inflammatory response and potentially reduced thromboembolic risk. This can be one of the aspects leading to pain relief.

By the fourth postoperative week, patients receiving DOT achieved an average knee flexion of 92.83°, nearly double that of the control group (53.83°), and complete extension (0° deficit) vs a persistent extension deficit of 5.67° in the control group. These correlations were statistically significant, indicating not only faster but also more complete functional recovery. Our results contradict a study on lower-limb pain (12), which showed DOT decreased lower-leg pain but did not significantly improve the range of motion. On the other hand, a study found that DOT reduced oedema and pain (20), and improved range of motion in early rehabilitation after knee arthroplasty (17). The mechanisms through which DOT may enhance ROM have not been fully explored. Possibly by reducing pain and inflammation early in the postoperative period, DOT facilitates more active and consistent participation in therapeutic exercises. Moreover, the therapy’s capacity to enhance interstitial fluid clearance and reduce periarticular swelling may decrease intra-articular pressure, which otherwise restricts joint movement.

The improvements in ROM observed in the DOT group may possibly be interrelated with the substantial reductions in postoperative knee swelling. Oedema is a well-recognized barrier to early joint mobilization, as excess intra- and periarticular fluid increases pressure, limits tissue extensibility, and contributes to pain and protective muscle inhibition (22). In this study, patients in the DOT group exhibited a significantly greater and more rapid decrease in knee circumference compared with the control group, with a reduction of nearly 5 cm over 4 weeks, vs just over 2 cm in the control group. These findings are consistent with prior research (16) using DOT after knee arthroplasty. Furthermore, the correlations observed between reduced swelling and improvements in CRP levels and ROM specifically in the DOT group suggest the therapy’s integrative effects on both local tissue recovery and systemic inflammatory status. These results suggest that the oedema-reducing properties of DOT are not only beneficial in isolation but also may play an enabling role in achieving early improvements in joint mobility and functional restoration.

Despite the results, this study is not without limitations. First, the sample size, while adequate to detect significant correlations, was relatively modest and limited to a single centre, which may affect the generalizability of the findings to broader populations. Second, while randomization was applied, blinding was not feasible for participants or therapists due to the nature of the intervention, and outcome assessors were not blinded, introducing a potential risk of performance bias. Additionally, the study focused exclusively on short-term outcomes within the first 4 weeks post-surgery; thus, the durability of the observed benefits of DOT over the medium and long term remains unknown. Objective measures, such as MRI or ultrasound imaging to assess intra-articular changes or fluid dynamics, were not included and could further validate the physiological effects of the therapy. Future research should aim to extend follow-up periods to assess functional recovery and complication rates. Moreover, studies exploring the cellular and molecular pathways influenced by DOT would help to strengthen the biological plausibility of its clinical effects. Future research should also include patient-reported outcome measures (PROMs), evaluate return to work and sports, analyse cost-effectiveness, assess feasibility for therapists, and examine potential reductions in hospitalization length.

The findings of this study suggest that incorporating DOT as an adjunct to a physiotherapy protocol may offer benefits in early postoperative rehabilitation following ACL reconstruction, particularly in reducing pain and swelling while enhancing joint mobility. Clinicians should consider DOT as a complementary modality to support recovery, especially in patients at risk of prolonged inflammation and a limited range of motion.

### Conclusions

In conclusion, adding DOT to kinesiotherapy enhances early postoperative recovery after ACL reconstruction. DOT, when added to physiotherapy, improved range of motion, reduced swelling, and lowered CRP and D-dimer within 4 weeks after ACL reconstruction. Larger studies with longer follow-up are needed to confirm these findings.

## References

[1] Sanders TL, Maradit Kremers H, Bryan AJ, Larson DR, Dahm DL, Levy BA, et al. Incidence of anterior cruciate ligament tears and reconstruction. Am J Sports Med 2016; 44: 1502–1507. 10.1177/0363546516629944 26920430

[2] Hunt ER, Jacobs CA, Conley CE-W, Ireland ML, Johnson DL, Lattermann C. Anterior cruciate ligament reconstruction reinitiates an inflammatory and chondrodegenerative process in the knee joint. J Orthop Res 2021; 39: 1281–1288. 10.1002/jor.24783 32558951

[3] Katagiri H, Nakamura K, Muneta T, Watanabe T, Miyatake K, Sekiya I, et al. Inflammatory and healing environment in synovial fluid after anterior cruciate ligament reconstruction: granulocytes and endogenous opioids as new targets of postoperative pain. Biochem Biophys Rep 2021; 26: 100981. 10.1016/j.bbrep.2021.100981 33997313 PMC8093890

[4] Spierings J, van Doeselaar M, Buenen M, Abinzano F, van der Steen M, Janssen R, et al. Inflammatory factors released by ACL reconstruction tendon grafts and their potential for macrophage polarization. Sci Rep 2025; 15: 19091. 10.1038/s41598-025-03788-w 40447668 PMC12125384

[5] Piedade SR, Leite Arruda BP, de Vasconcelos RA, Parker DA, Maffulli N. Rehabilitation following surgical reconstruction for anterior cruciate ligament insufficiency: what has changed since the 1960s? State of the art. J ISAKOS 2023; 8: 153–162. 10.1016/j.jisako.2022.10.001 36410671

[6] Eckenrode BJ, Carey JL, Sennett BJ, Zgonis MH. Prevention and management of post-operative complications following ACL reconstruction. Curr Rev Musculoskelet Med 2017; 10: 315–321. 10.1007/s12178-017-9427-2 28710739 PMC5577428

[7] Shelbourne KD, Patel DV. Treatment of limited motion after anterior cruciate ligament reconstruction. Knee Surg Sports Traumatol Arthrosc 1999; 7: 85–92. 10.1007/s001670050127 10223529

[8] Aliyev R. Klinische Wirksamkeit des Therapieverfahrens Tiefenoszillation bei Sportverletzungen. Sportverletzung ⋅ Sportschaden 2009; 23: 31–34. 10.1055/s-0028-1109216 19306234

[9] Boisnic S, Branchet M-C. Anti-inflammatory and draining effect of the Deep Oscillation® device tested clinically and on a model of human skin maintained in survival condition. Eur J Dermatol 2013; 23: 59–63. 10.1684/ejd.2012.1904 23420030

[10] von Stengel S, Teschler M, Weissenfels A, Willert S, Kemmler W. Effect of deep oscillation as a recovery method after fatiguing soccer training: a randomized cross-over study. J Exerc Sci Fit 2018; 16: 112–117. 10.1016/j.jesf.2018.10.004 30662504 PMC6323303

[11] Jahr S, Schoppe B, Reisshauer A. Effect of treatment with low-intensity and extremely low-frequency electrostatic fields (Deep Oscillation®) on breast tissue and pain in patients with secondary breast lymphoedema. J Rehabil Med 2008; 40: 645–650. 10.2340/16501977-0225 19020698

[12] Hausmann M, Ober J, Lepley AS. The effectiveness of deep oscillation therapy on reducing swelling and pain in athletes with acute lateral ankle sprains. J Sport Rehabil 2019; 28: 902–905. 10.1123/jsr.2018-0152 30526277

[13] Winkelmann ZK, Roberts EJ, Games KE. Acute effects and perceptions of deep oscillation therapy for improving hamstring flexibility. J Sport Rehabil 2018; 27: 570–576. 10.1123/jsr.2017-0044 28714788

[14] Villalba-Meneses F, Chaglla-Monge K, Almeida-Galárraga D, Cadena-Morejón C, Moreno-Calvo A, Marín J, et al. Evaluation of deep oscillation therapy for the treatment of lumbar pain syndrome using motion capture systems: a systematic review. J Bodyw Mov Ther 2024; 38: 180–190. 10.1016/j.jbmt.2024.01.010 38763561

[15] Koleva IB, Yoshinov BR, Asenova TA, Yoshinov RR. Physical analgesia: methods, mechanisms and algorithms for post-operative pain. In: Topics in Postoperative Pain. IntechOpen; 2023. 10.5772/intechopen.111590

[16] Christian M, Koenig R, Winkelmann Z, Games K. The effects of deep oscillation therapy for individuals with lower-leg pain. J Sports Med Allied Health Sci 2019; 4. 10.25035/jsmahs.04.03.03

[17] Vladeva E, Mihaylova M, Panayotova L. Deep oscillations – reducing edema and improving kinesiology in the early stages after knee joint arthroplasty. J IMAB - Annual Proceeding (Scientific Papers) 2021; 27: 3577–3581. 10.5272/jimab.2021271.3577

[18] Germolec DR, Frawley RP, Evans E. Markers of inflammation. Springer Nature Link; 2010. p. 53–73. 10.1007/978-1-60761-401-2_5 19967506

[19] Hopewell S, Chan A-W, Collins GS, Hróbjartsson A, Moher D, Schulz KF, et al. CONSORT 2025 statement: updated guideline for reporting randomised trials. BMJ 2025; 389: e081123. 10.1136/bmj-2024-081123 40228833 PMC11995449

[20] O’Brien CP, Watson A. Deep oscillation® therapy in the treatment of lateral epicondylalgia: a pilot randomized control trial. J Sports Med Doping Stud 2016; 6. 10.4172/2161-0673.1000180

[21] Koleva IB, Ioshinov BR, Yoshinov RD. Complex analgesia (infiltrations and deep oscillation) in patients with stump pain and phantom pain after lower limb amputation (double-blind randomised controlled trial of efficacy). J Adv Med Med Res 2017; 22: 1–17. 10.9734/JAMMR/2017/34198

[22] Hopkins JT, Ingersoll CD. Arthrogenic muscle inhibition: a limiting factor in joint rehabilitation. J Sport Rehabil 2000; 9: 135–159. 10.1123/jsr.9.2.135

